# Galantamine ameliorates hyperoxia-induced brain injury in neonatal mice

**DOI:** 10.3389/fnins.2023.890015

**Published:** 2023-06-22

**Authors:** Nahla Zaghloul, Naomi S. Cohen, Kamesh R. Ayasolla, Hsiu-Ling Li, Dalibor Kurepa, Mohamed N. Ahmed

**Affiliations:** ^1^Steele Children's Research Center, Division of Neonatology, Department of Pediatrics, University of Arizona, Tucson, AZ, United States; ^2^Neonatology Research Laboratory, Feinstein Institute for Medical Research, Northwell Health, Manhasset, NY, United States; ^3^Department of Neurology, Henry Ford Health, Detroit, MI, United States; ^4^Department of Physiology and Pharmacology, SUNY-Downstate Medical Center, New York, NY, United States

**Keywords:** hyperoxia induced brain injury, galantamine, acetylcholine esterase inhibitor, neuroinflammation, reactive oxygen species

## Abstract

**Introduction:**

Prolonged oxygen therapy in preterm infants often leads to cognitive impairment. Hyperoxia leads to excess free radical production with subsequent neuroinflammation, astrogliosis, microgliosis and apoptosis. We hypothesized that Galantamine, an acetyl choline esterase inhibitor and an FDA approved treatment of Alzheimer’s disease, will reduce hyperoxic brain injury in neonatal mice and will improve learning and memory.

**Methods:**

Mouse pups at postnatal day 1 (P1) were placed in a hyperoxia chamber (FiO_2_ 95%) for 7 days. Pups were injected IP daily with Galantamine (5 mg/kg/dose) or saline for 7 days.

**Results:**

Hyperoxia caused significant neurodegeneration in cholinergic nuclei of the basal forebrain cholinergic system (BFCS), laterodorsal tegmental (LDT) nucleus and nucleus ambiguus (NA). Galantamine ameliorated this neuronal loss. Treated hyperoxic group showed a significant increase of choline acetyl transferase (ChAT) expression and a decrease of acetyl choline esterase activity, thus increasing acetyl choline levels in hyperoxia environment. Hyperoxia increased pro-inflammatory cytokines namely IL -1β, IL-6 and TNF α, HMGB1, NF-κB activation. Galantamine showed its potent anti- inflammatory effect, by blunting cytokines surges among treated group. Treatment with Galantamine increased myelination while reducing apoptosis, microgliosis, astrogliosis and ROS production. Long term neurobehavioral outcomes at P60 showed improved locomotor activity, coordination, learning and memory, along with increased hippocampal volumes on MRI with Galantamine treated versus non treated hyperoxia group.

**Conclusion:**

Together our findings suggest a potential therapeutic role for Galantamine in attenuating hyperoxia-induced brain injury.

## Graphical abstract

**Graphical abstract:**
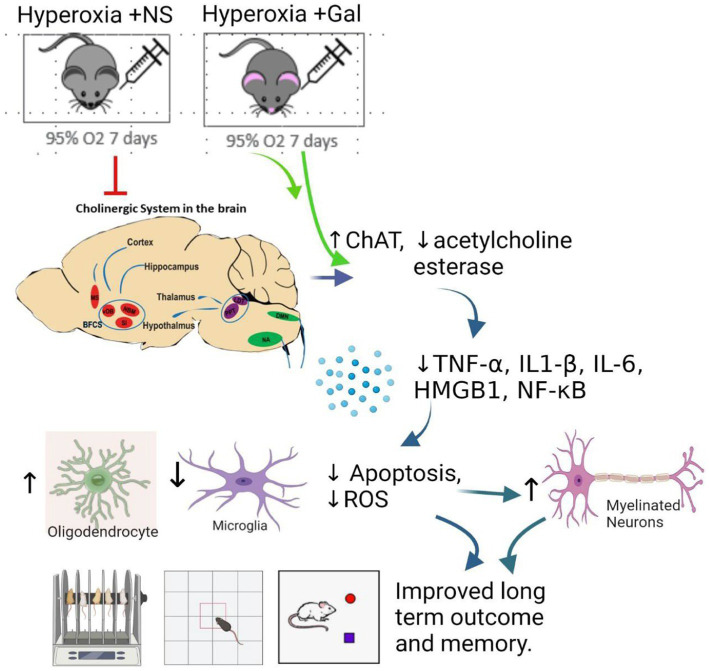
Galantamine anti-inflammatory and antioxidant benefits for neonatal hyperoxic brain injury

## Introduction

Brain injury in preterm infants is the leading cause of disability and death in children. Fetal development occurs under relative hypoxic conditions (PaO_2_: 25 mmHg) compared to extrauterine life (PaO_2_: 70 mm Hg) (Castillo et al., 2008). Premature infants’ tissues, including the brain, is exposed to relative hyperoxia compared to the intrauterine environment. This hyperoxia stress is further intensified by O_2_ supplementation used in treatment of the respiratory distress syndrome caused by lung immaturity (Felderhoff-Mueser et al., 2004).

In preterm infants, fluctuating or prolonged exposure to supraphysiological O_2_ levels may cause disruption of the neuronal circuitry resulting in encephalopathy of prematurity characterized by white matter injury (WMI) (van Tilborg et al., 2016). Furthermore, hyperoxia is one of the key risk factors for disabling cerebral palsy (Collins et al., 2001). Behavioral studies of very low birth weight premature infants exposed to hyperoxia show a heightened risk for anxiety, depression, and autistic like behaviors (Burnett et al., 2011; Pyhälä et al., 2014).

Exposure of neonate mice pups to 95% oxygen for 7 days is a well approved model of bronchopulmonary dysplasia with lung inflammation and impaired alveolarization (Auten et al., 2009). In rodents, hyperoxia causes neurocognitive impairment and memory deficits with smaller hippocampal sizes, similar to brain findings in ex-preterm infants (Ramani et al., 2013). Hyperoxia in rodents caused neuronal death, apoptosis, autophagy, oxidative stress, inflammation, altered neurotrophin growth factors and gene expression related to synaptic plasticity (Kaindl et al., 2006; Yiş et al., 2008; Ikonomidou and Kaindl, 2011; Reich et al., 2016). Rodents exposed to hyperoxia early in life developed hyperactivity and coordination deficits in adolescence, with cognitive impairment persisting into adulthood (Schmitz et al., 2012; Hoeber et al., 2016; Serdar et al., 2016).

Neonates have immature central nervous system with high metabolic demand. Their antioxidant defenses are immature leading to increase accumulation of free radicals (Perrone et al., 2015, 2016). Reactive oxygen species (ROS) inactivate enzymes, destroy DNA and proteins, eventually disrupting membrane function. ROS activates NF-κB leading to neuronal apoptosis and necrosis (Leviton et al., 2015). Free radicals induce mitochondrial membrane damage causing them to become leaky, releasing cytochrome C into the cytoplasm thus activating caspases which lead to severe injury to the developing brain (Love, 1999; Yuan and Yankner, 2000; Filomeni et al., 2003). Oxidative stress causes reactive astrogliosis, microgliosis and inflammation. Augmenting the antioxidant system by the over-expression of superoxide dismutase reduced hyperoxic brain injury in a neonate mouse model (Zaghloul et al., 2012). Since the use of O_2_ therapy in the neonatal period cannot be avoided, effective therapies to decrease the deleterious effect of hyperoxia are urgently needed.

Galantamine, a centrally acting acetylcholinesterase inhibitor, is widely used to treat cognitive deficits in Alzheimer’s diseases (Bullock, 2004). Galantamine crosses the blood brain barrier, stimulates cholinergic signaling and remains for an extended period of time (Noetzli and Eap, 2013). The brain cholinergic system plays a major regulatory role in memory and attention (Ballinger et al., 2016). Brain cholinergic signaling controls inflammation via the cholinergic anti-inflammatory pathway. This pathway controls cytokine production and inflammation through the efferent vagus nerve (Gallowitsch-Puerta and Pavlov, 2007). Impaired cholinergic signaling in murine sepsis survivors is a result of the interplay between systemic inflammation and neuroinflammation (Zaghloul et al., 2017). Galantamine minimizes oxidative damage in lymphocytes, using an *in vitro* model (Triana-Vidal and Carvajal-Varona, 2013). Galantamine decreased hypoxia-ischemia brain injury in a newborn rodent model (Furukawa et al., 2014). Physostigmine and donepezil, both cholinesterase inhibitors, reduced the deleterious neuronal effects of 80% oxygen exposure to 6-day old rat pups (Sifringer et al., 2013). However, the effects of Galantamine on hyperoxia induced brain injury has not been studied. In this work we investigate the neuroprotective role of galantamine treatment in neonatal mice who developed brain injury induced by hyperoxia exposure.

## Materials and methods

### Animal hyperoxia model

All procedures were performed in accordance with the NIH Guidelines on the care and use of vertebrate animals and approved by the Institutional Animal Care and Use Committee of the Feinstein Institute for Medical Research and the University of Arizona. Neonate C57BL6 mice at postnatal day 1 (P1) were placed in hyperoxia chamber system (BioSpherix, Lacona, NY, USA) which provided 95% normobaric oxygen for 7 days. Nursing mothers were switched every 24 h so that they are healthy and can lactate the pups. Animals were divided into 4 groups: room air (RA) + saline, RA + Galantamine, hyperoxia + saline, hyperoxia + Galantamine. Neonate mice were injected intraperitoneally with Galantamine at a dose of 5 mg/kg/day (dissolved in saline) at a volume of 0.1 mL. They were injected daily for 7 days starting from P1 after being placed in the hyperoxia chamber. Sham animals were injected IP with saline at 0.1 mL daily for 7 days starting from P1.

### Hematoxylin and eosin staining

Brain tissue was fixed on P14 in 4% paraformaldehyde for 24 h, processed, paraffin embedded, and sectioned sagittally at 6-μm-thickness. After deparaffinization, hematoxylin and eosin (H&E) staining was performed according to standard protocols. *N* = 5 mice per group.

### Immunohistochemistry

Animals were injected at P14 with a lethal dose of xylazine/ketamine and perfused transcardially with saline, and then 4% paraformaldehyde (PFA). Whole brain tissue was fixed in 4% PFA for 24 h, processed, paraffin embedded and sectioned sagittally and coronally at 6 μm thickness. For immunofluorescence, after deparaffinization and antigen retrieval, sections were incubated for 2 h at room temperature (RT) in TBS + 1% Triton-X + 10% donkey serum. Next, sections were incubated for 24 h at 4°C with primary antibodies, followed by 2 h incubation at RT with the appropriate secondary antibody with DAPI. All images were captured on a Zeiss confocal microscope (Carl Zeiss, Thornwood, NY, USA). The following primary antibodies were used to detect the following markers: Anti-ChAT (Millipore {1:100} Billerica, MA, USA); GFAP (Abcam {1:500} Cambridge, MA, USA); Iba1 (Wako {1:400}, Richmond, VA, USA); CNPase (Abcam {1:200} Cambridge, MA, USA); Olig2 (Santa Cruz Biotechnology{1:50} Dallas, TX, USA) and secondary antibodies (Species specific Cy3 and FITC {1:125} Jackson Immuno-research, Westgroove, PA).For negative control, sections were incubated with TBS + 1% Triton-X + 10% donkey serum for 24 h (no primary antibody added) followed by 2 h incubation with the appropriate secondary antibody with DAPI.

### Immunostaining analysis

Digital images were obtained using confocal software and then exported to Image J. Excitation and acquisition parameters were adjusted to fully eliminate pixel saturation and all images were collected under identical settings. Each section corresponds to 750 × 750 μm. The fluorescence intensity of each pixel was performed in 4 sections per mouse and 5 mice per group using image J.

Cell counting was performed using image J using plugins AnalyzeSkeleton (2D/3D). Skeletonized images are assessed for accuracy by creating an overlay of the skeleton and the original image. Cell counting was performed on 4 sections per animal (750 × 750 μm each) and 5 animals per group. Even though analysis was automated, all analysis was performed by one investigator for consistency who was blinded to the study group to eliminate bias.

### Cytokines assay

Proinflammatory cytokines IL-1β, IL-6, and TNF-α ELISAs were done on cortical tissue on postnatal day 8 using Quantikine ELISA kits (R&D Systems) that were used according to the manufacturer’s instructions. *N* = 5 animals/group.

Mouse HMGB1 chemiluminescence ELISA kit (Novus biologicals) was performed according to the manufacturer’s instructions. *N* = 5 animals/group.

### Western blot

Western blot was used to determine quantity of ChAT, phosphorylated p65 (marker of NF-κB activation) and P65 in cortical tissue homogenates on P8. After protein extraction, protein concentration was estimated using the Modified Lowry Protein Assay (Thermo Fisher Scientific, Rockford, IL, USA). Standard SDS-PAGE techniques were followed. After electrophoresis, proteins were transferred to a PVDF membrane using a Wet/Tank Blotting System (Bio-Rad, Hercules, CA, USA). Membranes were briefly washed, incubated with respective primary antibody in 5% BSA with PBST overnight. After washing, the membranes were incubated with HRP-conjugated secondary antibodies for 60 min, washed, processed using Amersham ECL detection systems (GE healthcare, Piscataway, NJ USA) and exposed to 8 × 10 Fuji x-ray Film. Density of ChAT band was presented as a ratio to Actin band density. Density of phosphoP65 was presented as a ratio to P65. The following primary antibodies were used: ChAT antibody (Millipore {1:1000} Billerica, MA, USA); phospho p65 (Cell Signaling Technology {1:500}, Danvers, MA, USA); P65 (Cell Signaling Technology {1:500}, Danvers, MA, USA); and anti-Beta- Actin protein (as an internal control) (Cell Signaling Technology {1:1000}, Danvers, MA, USA). Horseradish Peroxidase (HRP)-Conjugated Goat Anti-Rabbit and goat anti-mouse IgG conjugate were used for detection of rabbit and mouse primary antibodies, respectively, (Bio-Rad {1:5,000}, Hercules, CA, USA). *N* = 6 mice/group.

### Caspase 3 activity

Caspase 3 activity, as a marker for apoptosis, was measured on P8 with a Caspase-3 Colorimetric Assay (R&D Systems, Minneapolis, MN, USA). The assay was performed according to the manufacturer’s instructions, as described previously (Gerstner et al., 2008). The fluorescence was measured at the excitation wavelength of 360 nm, emission wavelength of 460 nm using a multiplate fluorescence reader (Biotek Instruments). The protein concentration was measured with a Pierce kit. Acetylated-7-Amino-4-methylcoumarin (AC-AMC) was used for obtaining the standard curve. Enzyme activity was calculated as picomoles per minute per milligram of protein.

### ROS assays

The DCFDA Intracellular ROS Assay (Abcam, Cambridge, MA, USA) was done per the manufacturer’s instructions on P8. Clear tissue lysates are placed in a 96-well cell culture plate and then pre-incubated with DCFH-DA, a standard substrate. The sample lysates were then added to the DCFH-DA. After an incubation period of 30 min, the samples were read on a standard fluorescence plate reader at 480 nm excitation/538 nm emission. The ROS levels in the samples was determined by comparison with the predetermined DCF standard curve (Kobayashi et al., 2016).

#### H_2_O_2_ assay

A colorimetric hydrogen peroxide detection kit [Enzo Life Sciences (ADI-907-015)] was used for the assay according to the manufacturer’s instructions. Standard concentrations of H_2_O_2_ were run along with the sample lysates. After incubation for 3 min, the absorbance was measured between 540 and 570. The samples’ H_2_O_2_ content was determined by comparison with the predetermined H_2_O_2_ standard curve (Wentworth et al., 2001).

### Acetylcholinesterase activity assay

Cortical brain tissue homogenates on P14 were prepared in a 20 mM Tris–HCl buffer (pH 7.3), containing 10 mM MgCl2, 50 mM NaCl, a protease inhibitor and zirconium beads using a bullet-blender homogenizer (Next Advance, Troy, NY, USA), according to the manufacturer’s recommendations. Homogenates were centrifuged (24,500 g), pellets were resuspended in equal volumes of 0.1 M NaHPO4 buffer (pH 10), then centrifuged again at (24,500 g), using the resultant supernatants (containing membrane bound AChE). AChE activity assay based on the widely used Ellman’s procedure (Guarini et al., 2004) and its modification was followed (Lee et al., 2010). A butyrylcholinesterase inhibitor was added to the reaction mix. The reaction was initiated by adding acetylthiocholine substrate, a thiolester. The thiocholine amount formed reflects AChE activity. The reaction mix color was read at 412 nm. Calculations using molar absorptivity (Ɛ) for thionitrobenzoate (TNB) were done at 412 nm. The results are expressed as mM of thiocholine released per minute at 25°C per 1 mL of lysate per 1 mg of protein. For positive control, dilute AChE was added instead of the supernatant and the same procedure was followed.

### Neurobehavioral testing and long-term outcome

Rotarod device (Columbus Instruments, Columbus, OH, USA) was performed at P60 to assess mice motor function, coordination and balance. Each session consisted of the average of three trials on the elevated accelerating rotarod beginning at 5 RPM, measuring the time the mouse was able to remain on the rod. At P60, mice were tested in an open field analysis (San Diego Instruments, San Diego, CA, USA). Mice had one 30 min training session before the beginning of testing, to adapt to the testing chamber. Open field data was digitally recorded for 30 min and then analyzed by Noldus Ethovision tracking software (Hartman et al., 2009). Beam breaks were recorded in the *x*, *y*, and *z* planes and averaged across groups. Novel object recognition test was performed on P60 to evaluate learning and memory. Mice were adapted to empty box once a day for 3 days, then adapted to a box containing 2 identical objects once a day for 3 days. One of the objects in the box was replaced by a new one for which they were adapted also once a day for 3 days. Then testing began. When mice sniffed or touched the objects, but not climbed over the objects, it was considered an effective exploration. The exploration time was recorded by two blinded observers to treatment/exposure allocation. The “recognition index” was out according to a formula: (exploring novel object time- exploring familiar object time)/(total exploration time for novel and familiar objects) (Mei et al., 2020). *N* = 25 mice/group.

### Statistical analysis

For all statistical tests, Graph Pad Prism 8 software (La Jolla) was used. Statistical analysis of mean differences between groups was performed by using student *t*-test or one-way ANOVA, followed by a Bonferroni post-hoc analysis. All *p* values and *n* values are indicated in figure legends.

Two hundred twenty total pups were used for all experiments. Male/female ratio is 6:5. No sex difference was found in the studies.

## Results

Oxygen therapy is a double edge sword for preterm infants. It is essential for the treatment of their respiratory distress, at the same time causing bronchopulmonary dysplasia, cognitive dysfunction and free radical injury to all organs (Mohamed et al., 2020). We hypothesized that galantamine would ameliorate this hyperoxic brain injury, while improving cognition and memory in a neonate mouse model of hyperoxia (Zaghloul et al., 2012). There was no mortality in any of the mice groups.

**Figure 1:**
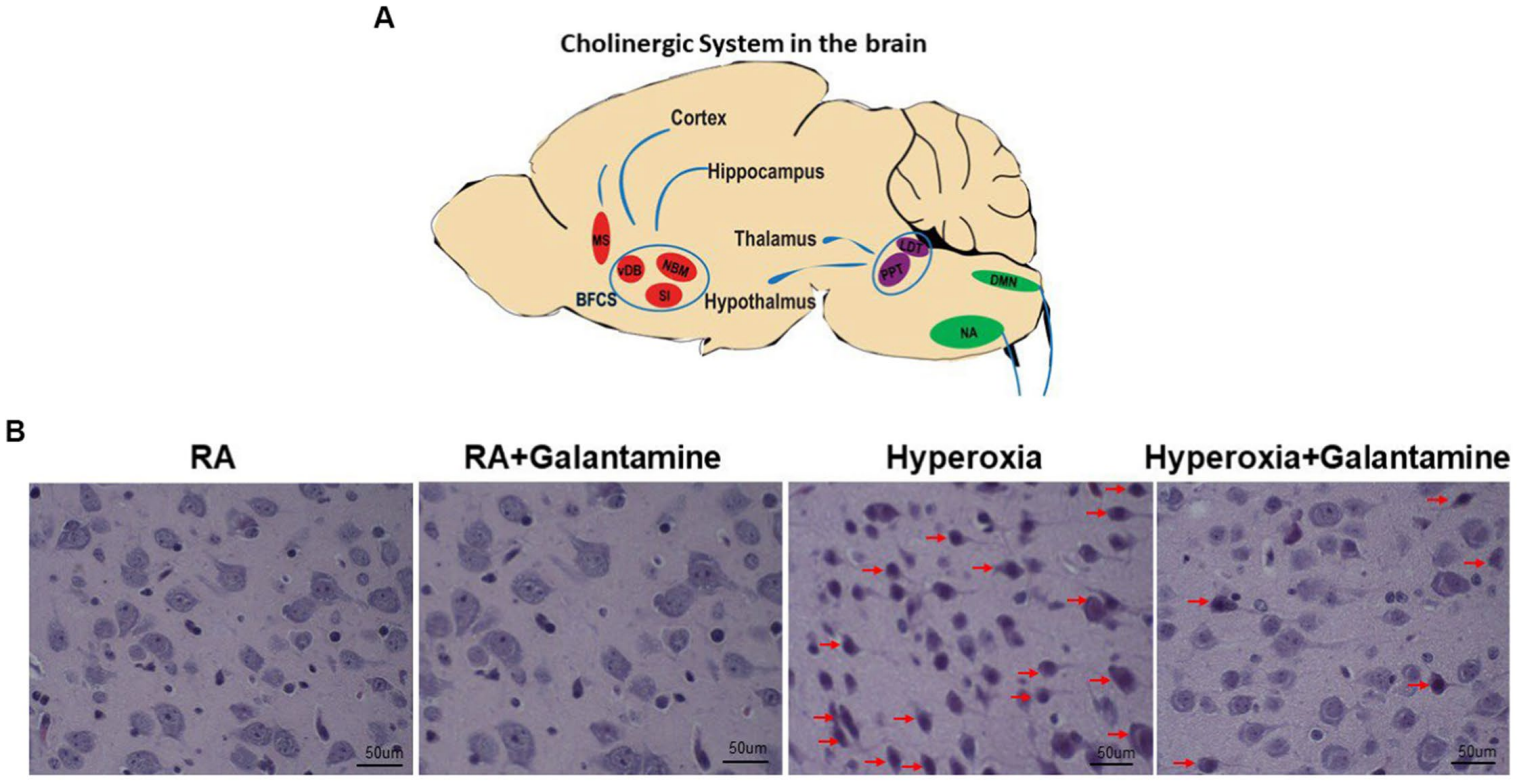
Galantamine rescues cholinergic neurons from hyperoxia induced neurodegeneration. **(A)** Simplified schematic diagram of the Cholinergic system of the brain. Sagittal view from anterior (1) to posterior (6). Medial septum (MS). Basal forebrain cholinergic system (BFCS) which consist of the vertical limbs of the diagonal band of Broca (vDB), substantia innominate (SI) and nucleus basalis of Meynert (NBM), projecting to the cortex and hippocampus. Brain stem cholinergic nuclei: Peduncloppontine tegmental (PPT) nucleus and Laterodorsal tegmental (LDT) nucleus. Both PPT and LDT innervate thalamus, hypothalamus and hindbrain. Nucleus amibiguus (NA) Dorsal motor nucleus of the vagus (DMN). NA and DMN provide cholinergic projection with the vagus nerve to the periphery (heart, lung, liver, and other organs supplied by the vagus nerve). **(B)** H&E stain on P14 of the basal forebrain cholinergic system showing cholinergic nuclei of room air (RA), hyperoxia and hyperoxia+ galantamine groups. Red arrows indicate degenerating cholinergic nuclei. Hyperoxia causes extensive neurodegeneration of cholinergic nuclei which is rescued by galantamine administration. Magnification 40x. Scaler bar = 50 μm. *N* = 5 animals/group & 4 sections/animal.

To better elucidate the central cholinergic actions of galantamine, we show a simplified schematic diagram of the cholinergic system of the brain (Figure 1A). A sagittal section of the brain with cholinergic nuclei from anterior to posterior is provided in Figure 1A. Medial septum (MS). Basal forebrain cholinergic system (BFCS) which consist of the vertical limbs of the diagonal band of Broca (vDB), substantia innominate (SI) and nucleus basalis of Meynert (NBM), projecting to the cortex and hippocampus. Brainstem cholinergic nuclei: Pedunculopontine tegmental (PPT) nucleus and Laterodorsal tegmental (LDT) nucleus. Both PPT and LDT innervate thalamus, hypothalamus and hindbrain. Nucleus amibiguus (NA) and Dorsal motor nucleus of the vagus (DMN). NA and DMN provide cholinergic projection with the vagus nerve to the periphery (heart, lung, liver and other organs supplied by the vagus nerve).

### Galantamine rescues cholinergic neurons from hyperoxia induced neurodegeneration

Hyperoxia causes cortical, hippocampal and cerebellar neuronal damage and degeneration in the form of apoptosis and necrosis (Gerstner et al., 2008). H&E staining of cholinergic cells showed neuronal degeneration of the Basal forebrain cholinergic system in hyperoxic non-treated group (red arrows in Figure 1B). Galantamine treatment rescues cholinergic neurons from hyperoxia induced neuronal degeneration as shown in Figure 1B. There is no difference between RA+ saline and RA+ galantamine group.

H&E staining of cholinergic cells showed neuronal degeneration of the Lateral Dorsal Tegmental nuclei (LDT, upper panel) and Nucleus Ambiguus (NA, lower panel) in hyperoxic non-treated group (red arrows in Supplementary Figure S1). Galantamine treatment rescues cholinergic neurons from hyperoxia induced neuronal degeneration (Supplementary Figure S1).

### Galantamine rescues cholinergic nuclei from hyperoxia induced neuronal loss

**Figure 2:**
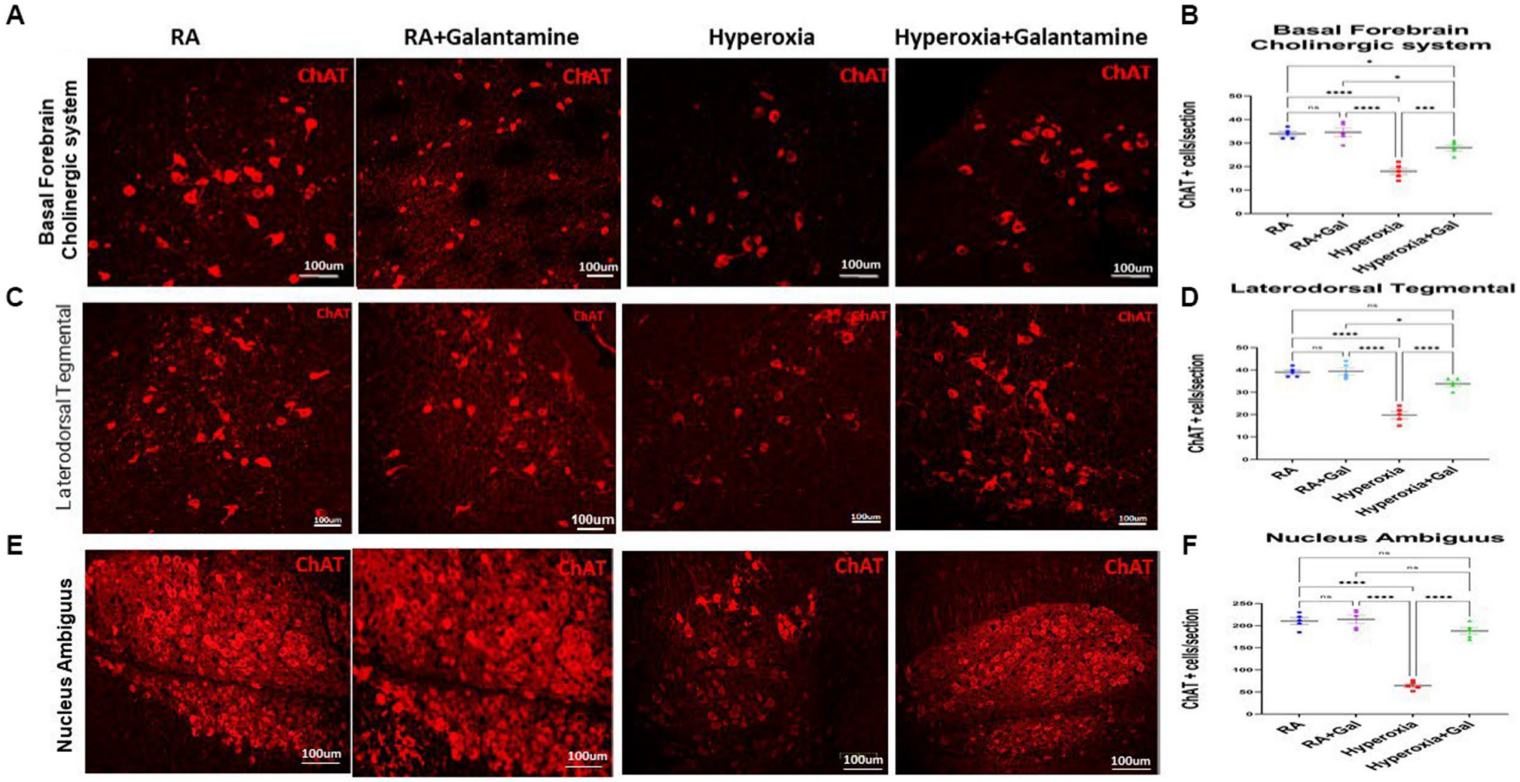
Galantamine rescues cholinergic nuclei from hyperoxia induced neuronal loss. **(A)** Immunohistochemistry of choline acetyltransferase containing cells (ChAT) of the Basal forebrain cholinergic system (BFCS) at P14 in all study groups. Scale bar = 100 μm. **(B)** Quantification of ChAT positive cells per section in BFCS. *N* = 5 animals/group & 4 sections/animal. Scatter dot plot showing mean ± SE. *p ≤ 0.05, ****p ≤ 0.0001. **(C)** Immunohistochemistry of choline acetyltransferase containing cells (ChAT) of Laterodorsotegmental (LDT) nucleus at P14 in all study groups. Scale bar = 100 μm. **(D)** Quantification of ChAT positive cells per section in LDT nucleus. *N* = 5 animals/group & 4 sections/animal. Scatter dot plot showing mean ± SE., ****p ≤ 0.0001. **(E)** Immunohistochemistry of choline acetyltransferase containing cells (ChAT) of Nucleus ambiguous nucleus (NA) at P14 in all study groups. Scale bar = 100 μm. **(F)** Quantification of ChAT positive cells per section in NA nucleus. *N* = 5 animals/group & 4 sections/animal. Scatter dot plot showing mean ± SE., ****p  ≤ 0.0001.

Hyperoxia causes cholinergic neuronal damage and degeneration which leads to apoptosis and necrosis and eventually neuronal loss (Reich et al., 2016). Examining the cholinergic system by assessing cholinergic neuronal cell number was done by immunofluorescent staining for Choline acetyltransferase (ChAT), the enzyme that is responsible for the synthesis of acetyl choline (Ach), the main neurotransmitter for the parasympathetic system. We show that hyperoxia caused a significant loss of ChAT positive cells in the BFCS, while treatment with Galantamine, prevented loss of ChAT positive neurons after hyperoxia exposure (Figures 2A,B). Hyperoxia also caused a significant loss of ChAT positive cells in LDT (Figures 2C,D), and NA (Figures 2E,F), while Galantamine treatment in this hyperoxic setting rescued ChAT positive neuron in the BFCS, LDT and NA to the RA numbers. There was no difference between the 2 RA groups in all three cholinergic nuclei (Figure 2). The loss of ChAT positive neurons leads to a decrease in Ach and thus parasympathetic innervation to the areas innervated by the BFCS, LDT and NA.

### Galantamine ameliorates hyperoxia induced reductions in cholinergic brain activity

**Figure 3:**
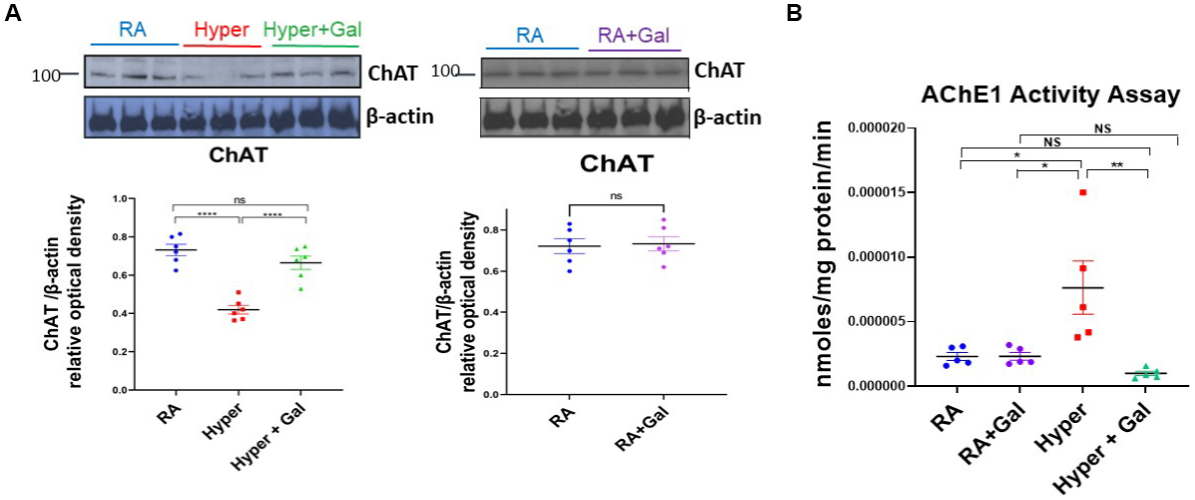
Galantamine ameliorates hyperoxia induced reductions in cholinergic brain activity. **(A)** ChAT western blot of cortical protein lysate at P14 in RA, Hyperoxia and Hyperoxia +Galantamine groups, represented as a ratio to β -actin protein. Scatter dot plot densitometry quantification analysis represented as ChAT/β -actin relative optical density showing mean ± SE., ****p ≤ 0.0001. ChAT western blot of cortical protein lysate in RA + Saline group as compared to RA + Galantamine group represented as ChAT/β - actin relative optical density showing mean ± SE., P is ns. *N* = 6 animals/group. **(B)** Acetylcholinesterase activity assay of cortex presented as nmol/mg protein/min at P14 in all study groups. *N* = 5 animals/group Scatter dot plot showing mean ± SE., *p ≤ 0.05, **p < 0.01.

Hyperoxia significantly decreases ChAT protein expression in the BFCS as shown by western blot study, while ChAT protein levels were preserved in hyperoxia group treated with Galantamine, with ChAT protein levels equivalent to RA group (Figure 3A). ChAT protein expression levels (Figure 3A) coincides with ChAT neuronal staining in the BFCS (Figures 2A,B). Acetylcholine esterase breaks down acetylcholine. As acetylcholine esterase activity increases, ChAT protein expression decreases. To explore if that is in fact the reason for the decreased in ChAT protein expression, we analyzed BFCS acetylcholine esterase activity. In hyperoxia group, acetylcholine esterase activity was significantly increased in comparison to both RA groups and Galantamine hyperoxia treated group. Galantamine treated hyperoxia group had similar acetylcholine esterase levels to both RA groups (Figure 3B).

### Galantamine has an anti-inflammatory effect on glia in hyperoxic environment

**Figure 4:**
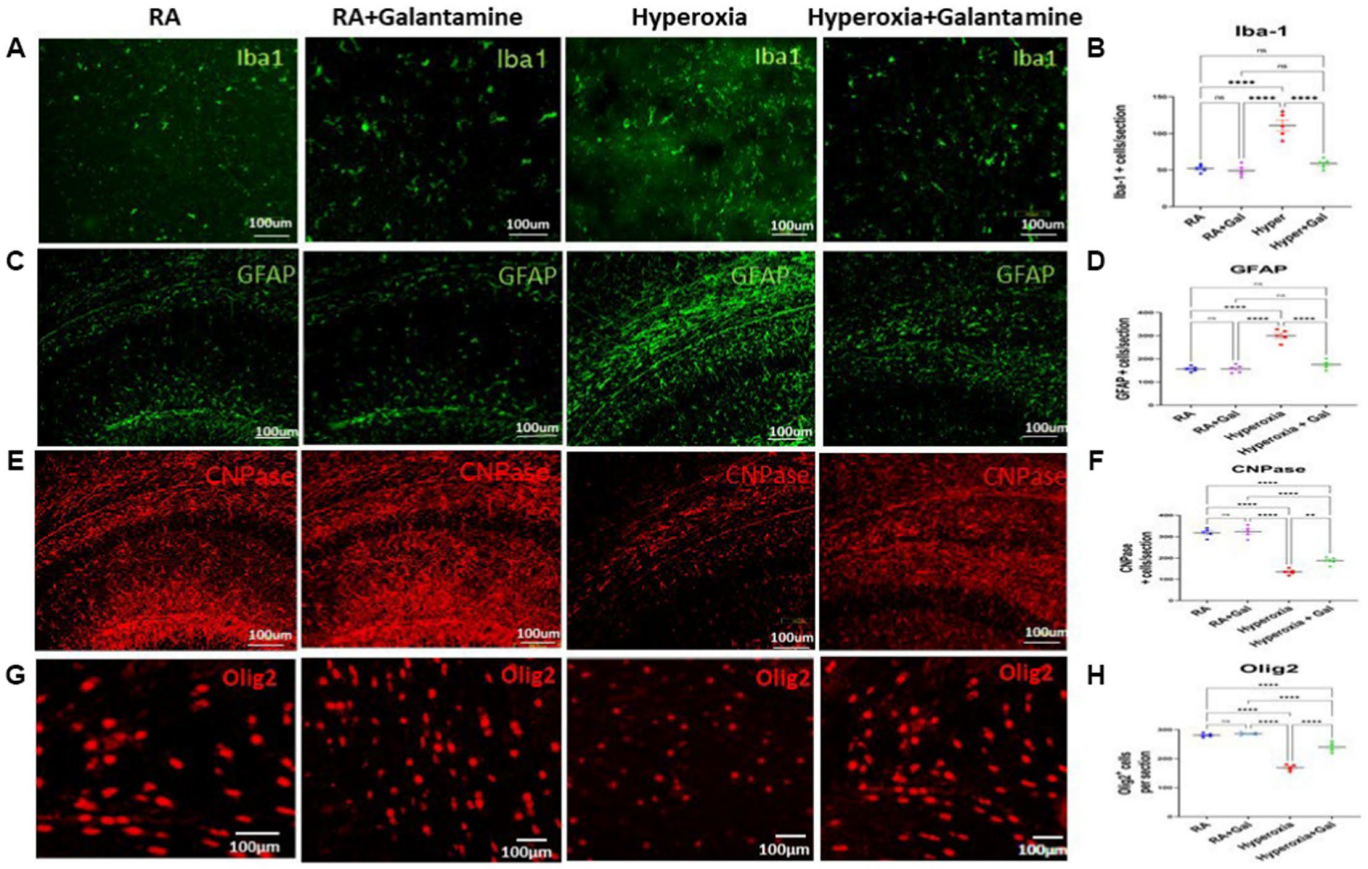
Galantamine has an anti-inflammatory effect on glia in hyperoxic environment **(A)** Immunohistochemistry of microglia (Iba1 in green) in the periventricular white matter area on P14 in all study groups. Scale bar = 100 μm. **(B)** Quantification of Iba-1 positive cells per section. *N* = 5 animals/group & 4 sections/animal. Scatter dot plot showing mean ± SE. ****p ≤ 0.0001. **(C)** Immunohistochemistry of astrocytes (GFAP in green) in the CA1 area of the hippocampus and dentate gyrus on P14 in all study groups. Scale bar = 100 μm. **(D)** Quantification of GFAP intensity as fold change where RA = 1. *N* = 5 animals/group & 4 sections/animal. Scatter dot plot showing mean ± SE. ****p ≤ 0.0001. **(E)** Immunohistochemistry of Pre-oligodendrocytes (CNPase in red) in the CA1 area of the hippocampus and dentate gyrus on P14 in all study groups. Scale bar = 100 μm. **(F)** Quantification of CNPase intensity as fold change where RA = 1. *N* = 5 animals/group & 4 sections/animal. Scatter dot plot showing mean ± SE. ****p ≤ 0.0001. **(G)** Immunohistochemistry of oligodendrocytes (Olig-2 in red) in the periventricular white matter area on P14 in all study groups. Scale bar = 100 μm. **(H)** Quantification of Olig-2 positive cells per section. *N* = 5 animals/group & 4 sections/animal. Scatter dot plot showing mean ± SE. **p < 0.001, ****p ≤ 0.0001.

Microgliosis and microglial activation potentiates neuroinflammation by releasing pro- inflammatory cytokines (Zaghloul and Ahmed, 2017). Hyperoxia resulted in microgliosis (indicated by Iba1 staining) especially in the white matter where microglia are more abundant. Galantamine treated hyperoxia group shows a significant reduction in microgliosis in the periventricular white matter as compared to the hyperoxia group. No difference was found in microglial number between Galantamine treated hyperoxia group and RA groups (Figures 4A,B). There is also no difference in microglial numbers between RA and RA + Galantamine groups.

Astrogliosis causes blood brain barrier disruption leading to formation of the glial scar (Zhou et al., 2020). Hyperoxia caused significant astrogliosis shown in the CA1 area of the hippocampus and dentate gyrus as indicated by the increase in GFAP staining in the hyperoxia group hippocampal region compared to the RA groups. Galantamine treatment significantly reduced astrogliosis induced by hyperoxia in comparison to non-treated hyperoxia group (Figures 4C,D). Galantamine administration to RA group has no effect on astrocytes.

Myelination of the hippocampus leads to improvements of both learning and memory. Galantamine treatment to RA group did not alter pre-oligodendrocyte or oligodendrocyte numbers. We show a significant reduction in pre-oligodendrocytes in the CA1 area of the hippocampus and dentate gyrus indicated by CNPase staining in the hyperoxia group compared to the RA group. Pre-oligodendrocytes were preserved in the hyperoxia group treated with Galantamine (Figures 4E,F). Oligodendrocytes indicated by Olig −2 staining in the periventricular white matter area were also significantly decreased in the hyperoxia group as compared to both RA groups. Galantamine reduced oligodendrocytes loss caused by hyperoxia, thus ameliorating white matter loss (Figures 4G,H).

### Galantamine attenuates neuro-inflammation, apoptosis, and oxidative stress in hyperoxic environment

**Figure 5:**
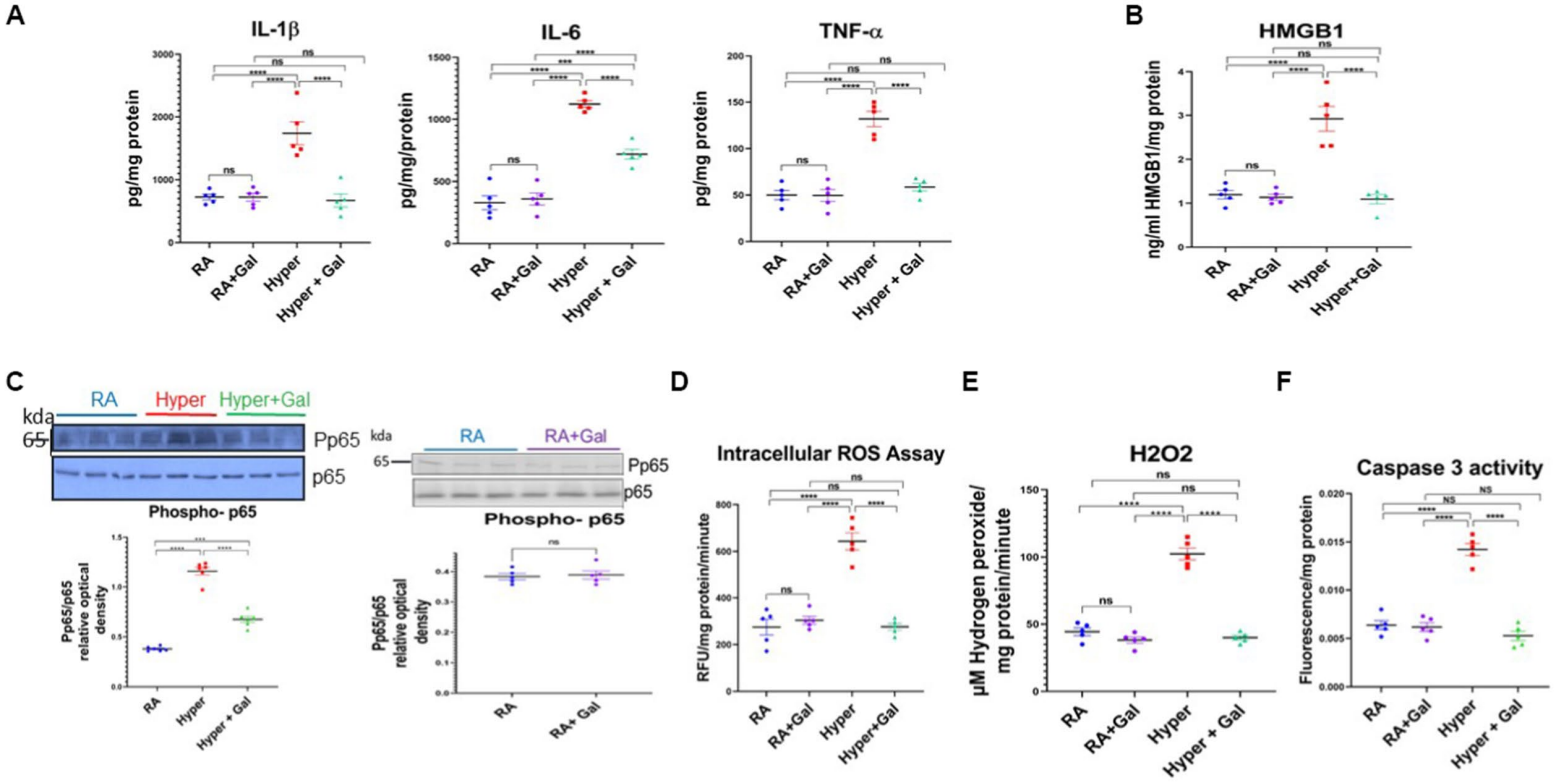
Galantamine attenuates neuro-inflammation, apoptosis and oxidative stress in hyperoxic environment. **(A)** Brain pro-inflammatory cytokine levels IL1β, IL-6, TNF α measured by ELISA from cortical brain lysates on P8 in all study groups. *N* = 5 animals/group. Scatter dot plot showing mean ± SE. ****p ≤ 0.0001. **(B)** Brain high mobility group box 1 (HMGB1) assay by ELISA from cortical brain lysates on P8 in all study groups. *N* = 5 animals/group. Scatter dot plot showing mean ± SE. ***p < 0.001, ****p ≤ 0.0001. **(C)** Brain Phosphorylated P65 western blot represented as a ratio to p65 protein at P8 of cortical brain lysates in RA, Hyperoxia, Hyperoxia + Galantamine. Brain Phosphorylated P65 western blot represented as a ratio to p65 protein at P8 of cortical brain lysates in RA + Saline and RA + Galantamine *N* = 6 animals/group. Scatter dot plot showing mean ± SE. P ns, ***p < 0.001, ****p ≤ 0.0001. **(D)** DCFDA intracellular ROS assay, measuring superoxide and hydroperoxyl expressed as relative florescence units (RFU) per mg protein per minute in neonate mouse cortex on P8 in all study groups. *N* = 5 animals/group. Scatter dot plot showing mean ± SE. ****p ≤ 0.0001. **(E)** Hydrogen peroxide ROS assay measuring μM hydrogen peroxide per mg protein per minute in neonate mouse cortex on P8 in all study groups. *N* = 5 animals/group. Scatter dot plot showing mean ± SE. ****p ≤ 0.0001. **(F)** Activated Caspase 3 activity by ELISA measuring apoptosis in neonate mouse brain cortex tissue on P8 of cortical brain lysates in all study groups. *N* = 5 animals/group. Scatter dot plot showing mean ± SE. ****p ≤ 0.0001.

Proinflammatory cytokines namely IL-1β, IL-6, TNFα, were significantly increased in the cortex of hyperoxic pups as compared to the RA groups. Galantamine treated hyperoxia group had a significant reduction in the levels of proinflammatory cytokines as compared to hyperoxia group (Figure 5A). Brain high mobility group box 1 (HMBG1) is one of the Damage Associated Molecular Patterns (DAMP) secreted by microglia and is an initiator and amplifier of neuroinflammation (Gou et al., 2020). HMBG1 was significantly increased in hyperoxia group as compared to RA group. Galantamine treated hyperoxia group had a significant decline in HMBG1 as compared to hyperoxia group (Figure 5B). These finding indicate that Galantamine attenuates neuro-inflammation. Hyperoxia exposure caused a significant increase of phosphorylated p65/p65, indicating NF-KB activation when compared to RA groups. Galantamine treated hyperoxia group had a significantly lower phosphorylated p65/p65 when compared to hyperoxia group (Figure 5C). There is no difference in cytokine levels, HMGB1 or NF-KB activation between the two RA groups. These findings highlight the anti-inflammatory role of Galantamine. Compared to the two RA groups, hyperoxia caused a significant increase in ROS levels assessed by both Oxiselect intracellular ROS assay, (measures intracellular superoxide and hydroperoxyl radical), and H_2_O_2_ assay (measures hydrogen peroxide). Galantamine treated hyperoxia group had significantly lower ROS levels than hyperoxic non-treated group (Figure 5D). These data support the anti-oxidative role of Galantamine.

Increase in ROS production overwhelms the cellular antioxidant capacity and results in damage to the DNA, proteins and lipids leading to apoptosis and necrosis (Thannickal and Fanburg, 2000). Thus, caspase 3 activity was measured to assess apoptosis in all studied groups. Caspase 3 activity was significantly increased in the hyperoxia group as compared to the RA groups. Galantamine treated hyperoxia group had a significantly lower caspase 3 activity when compared to hyperoxia group, with levels similar to RA group (Figure 5F). Galantamine treatment to the RA group had no difference in ROS levels or apoptosis, in comparison to RA saline group. These findings suggest anti-apoptotic activity of galantamine.

To evaluate late stages of neuronal apoptosis and neuronal necrosis, we performed Tunel staining. We examined Tunel colocalization with ChAT positive cholinergic nuclei in the BFCS as an indication of cholinergic neuronal necrosis. Tunel staining was significantly increased in the hyperoxia BFCS ChAT positive nuclei as compared to the RA groups. Galantamine treated hyperoxia group had a significantly lower Tunel staining when compared to hyperoxia group, thus providing protection against cholinergic neuronal necrosis (Supplementary Figure S2).

### Galantamine improves long term neurodevelopmental outcomes in hyperoxia

**Figure 6:**
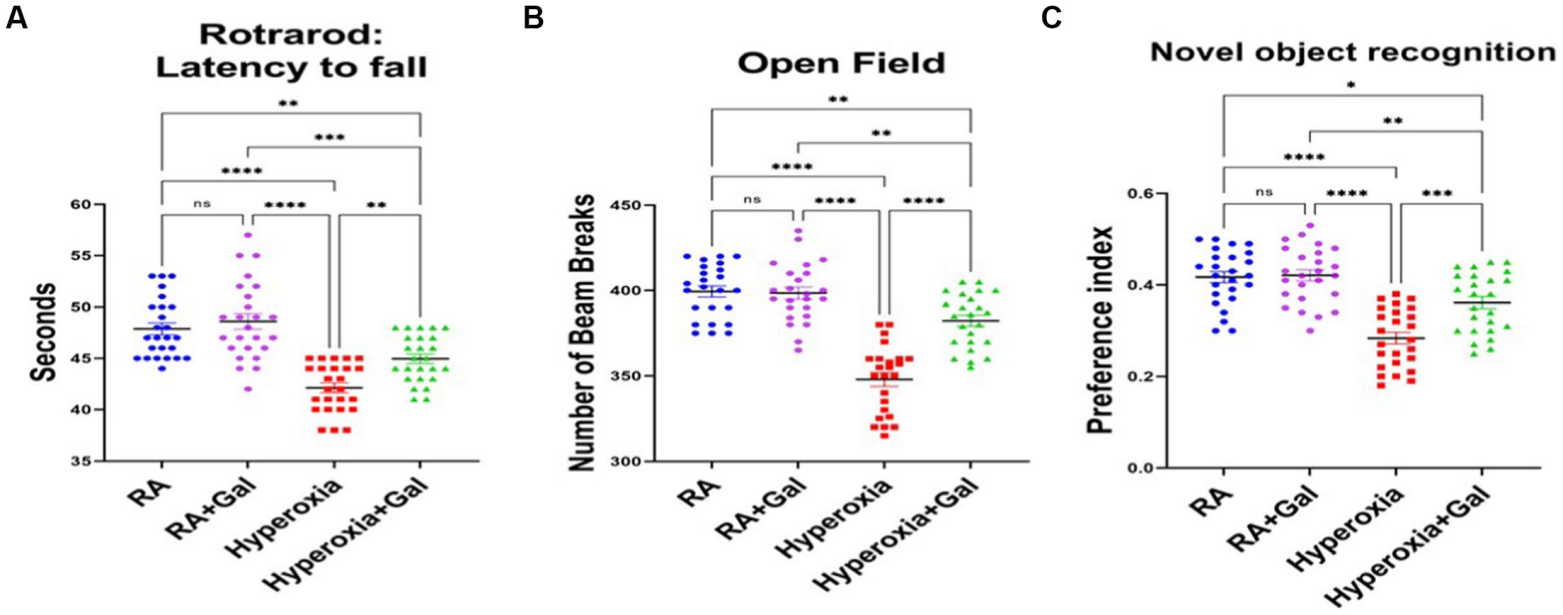
Galantamine improves long term neurodevelopmental outcomes in hyperoxia. **(A)** Testing of latency to fall in seconds by rotarod for coordination assessment at P60. in all study groups. *N* = 25 animals/group. Scatter dot plot showing mean ± SE. ***p ≤ 0.001, ****p ≤ 0.0001. **(B)** Number of beam breaks in the open field test as an assessment of motor function at P60 in all study groups. *N* = 25 animals/group. Scatter dot plot showing mean ± SE. **p ≤ 0.01, ****p ≤ 0.0001. **(C)** Showing preference index of novel object recognition as a measure of memory at P60 in all study groups. *N* = 25 animals/group. Scatter dot plot showing mean ± SE. **p ≤ 0.01, ***p ≤ 0.001, ****p ≤ 0.0001.

To investigate if the improvement in histopathological findings, cholinergic activity and oligodendrocyte numbers, along with reduced neuro-inflammation and oxidative stress, translate to improvement in neurodevelopmental outcomes, neurobehavioral analysis was performed at P60. On rotarod, hyperoxia group had impaired coordination as they had a more tendency to fall from the rotarod, while Galantamine treated hyperoxia group had less tendency to fall, thus improved co-ordination than the hyperoxic group (Figure 6A). Open field analysis shows a significantly reduced number of beam breaks, indicating worsening locomotion in hyperoxia versus hyperoxia Galantamine mice group (Figure 6B). Preference index for the novel object in the novel object recognition test was significantly increased in Galantamine treated hyperoxia group than non-treated hyperoxia group (Figure 6C). Mice with better recognition memory spend more time exploring a novel object than a familiar one. Galantamine administration to RA showed no significant difference to RA group in all the above neurobehavioral outcomes measured. This data indicated improved memory function especially recognition memory in Galantamine treatment hyperoxia group versus non treated one.

Since we showed that Galantamine improves memory function, therefore we aimed to measure hippocampal volume at P60, to correlate with the novel object recognition test. Hyperoxia significantly decreases hippocampal volumes measured on T2 weighted images. Galantamine treated hyperoxia group had larger hippocampal volumes as compared to hyperoxia alone (Supplementary Figure S3). RA and RA + Galantamine groups had no difference in hippocampal volumes. Hyperoxia can cause severe and permanent retinal degeneration. We show that in hyperoxia, the hyaloid artery was hyperplastic and extended onto the retina near the optic nerve head and formed a thick distinct capsule on the posterior lens surface. Outer retinal degeneration with elevated and disrupted retinal layers and neovascularization was evident in hyperoxic pups. In Galantamine treated pups that undergone hyperoxia, the hyaloid artery was less hyperplastic, also formed a capsule on the posterior lens surface, and the outer retinal cell disruption and neovascularization was less severe (Supplementary Figure S4).

## Discussion

Our study provides strong evidence that supports the protective role of galantamine in hyperoxic brain injury in neonate mice. Galantamine is a reversible and competitive acetyl choline esterase inhibitor and a positive allosteric modulator of nicotinic acetylcholine receptors. Galantamine inhibits the AChE from dissociating acetyl choline, therefore increasing the neurotransmitter acetyl choline level and duration of action (Colović et al., 2013). In hyperoxic mice, we showed a significant increase of AChE activity as compared to RA and galantamine treated hyperoxia groups (Figure 3B). This finding was accompanied by a significant reduction of ChAT expression as shown by Western Blot assay and immunostaining (Figures 2, 3A).

Galantamine potent anti-inflammatory properties is mainly attributed to its ability to increase acetylcholine level and activity, a known anti-inflammatory neurotransmitter, through the inhibition of AChE. Another anti-inflammatory mechanism of galantamine is inhibition of microgliosis, astrogliosis, pro-inflammatory cytokines and NF-κB activation induced by hyperoxia.

Premature infants are at high risk for infection/inflammation as they are often born to mothers with chorioamnionitis. They have very low innate and acquired immunity as compared to full term infants (Villamor-Martinez et al., 2020).

We showed an overall reduction in inflammatory response related to hyperoxia stress in the galantamine treated group through significant reduction of different inflammatory cytokines including IL-1β, IL-6, TNFα, pNF-κB, and HMGB1 compared to non-treated hyperoxia group (Figures 5A–C). Our findings support the anti-inflammatory properties of galantamine, which were also shown previously, in neonatal hypoxic ischemic rats (Furukawa et al., 2014). Galantamine, therefore restores the normal milieu for brain development and brain cell proliferation and maturation.

The immature brain is extremely vulnerable to ROS (produced by hyperoxia) due to high oxygen consumption rate, high concentrations of unsaturated fatty acids, low levels and activity of antioxidants, and large amount of susceptible immature cells (Collins et al., 2001). Galantamine acts as an antioxidant by decreasing intracellular ROS levels through decreasing ROS overproduction and scavenging of already produced ROS (Figure 5D).

Galantamine increases acetylcholine levels and phosphorylates serine–threonine protein kinase. Through stimulation of phosphoinositide 3-kinase and increased expression of protective protein Bcl-2, galantamine decreases overproduction of ROS (Mei et al., 2020). Galantamine scavenges reactive oxygen species by inhibiting the activation of P2X7 receptors, stabilizing mitochondrial membrane potential and preventing membrane fluidity disturbance (Tsvetkova et al., 2013). Oxygen therapy cannot be avoided in neonatal intensive care. Therefore, when oxygen supplementation is required, in addition to developing appropriate monitoring systems, protective and/or regenerative strategies are highly warranted.

Neuroinflammation and excessive ROS causes pre-oligodendrocyte injury and death leading to eventual white matter loss (Zaghloul et al., 2017) Pre-oligodendrocytes are the most sensitive and vulnerable to oxidative stress and neuroinflammation. As anticipated, reduction of the free radicals and attenuation of the inflammatory response through galantamine administration plays significant role in preserving oligodendrocytes and myelination.

Oxidative stress induces direct brain cell injury in both grey and white matter. Hyperoxia induces upregulation of inducible nitric oxide synthetase (iNOS) mRNA which is mainly synthesized by microglia (Hoehn et al., 2003). Free radicals and increased production of nitric oxide (NO), both induce cell death and DNA fragmentation in brain tissue (Platt and Riedel, 2011). In our hyperoxic neonate group, there was significant apoptosis compared to the RA groups (Figure 5F). Our results show that administration of galantamine during hyperoxia led to a significant reduction of ROS levels (Figure 5D) and inhibition of apoptosis as shown by reduction of caspase 3 activity (Figure 5E) as well as a protection of cholinergic neurons from hyperoxia induced neurodegeneration (Figure 1B). Further studies investigating the effect of hyperoxia and galantamine administration on neuronal and glial cell proliferation and maturation are needed.

Brain cholinergic system plays a significant role during neonatal brain development by modulating neurogenesis, gliogenesis, synaptic plasticity, attention, learning, memory, REM sleep (Platt and Riedel, 2011) and the control of movements. Disruption of the cholinergic system during brain development, leads to adverse effects on brain development in neonates especially premature infants (Abreu-Villaça et al., 2011). Hyperoxia leads to neuronal and glial cell death, resulting in white and grey matter brain injury observed in preterm infants. During the critical phase of brain maturation, hyperoxia can alter developmental processes, disrupting neural plasticity and myelination (Reich et al., 2016). Our study had similar findings to those seen in preterm infants, showing significant reduction of myelination (Olig-2 staining) with hyperoxia and myelin preservation when galantamine is administered to hyperoxia group.

In our study, galantamine administration during hyperoxia exposure leads to preservation of ChAT levels. This rescue of ChAT+ cells in the BFCS (Figure 2A) which gives cholinergic innervation to neocortex and hippocampus (Figure 1A) leads to improvement of learning and memory as shown by increased preference index for the novel object in the novel object recognition test (Figure 6C). The rescue of ChAT in the LDT which supplies the thalamus, hypothalamus and hindbrain improves motor function (Figure 6B) and REM sleep. All cholinergic nuclei contribute a significant role in reducing inflammation (Figures 5A–C), improving myelination (Figures 4G,H) and memory (Figure 6C). Hippocampal volume is significantly preserved and protected from the damaging effects of hyperoxia through increasing the BFCS’s cholinergic innervation to the hippocampus by Galantamine administration (Supplementary Figure S3). This, along with decreased neuro-inflammation, neuronal apoptosis and ROS, led to improved hippocampal volume and memory function. Restoring cholinergic system integrity, as well as activity after galantamine administration to the hyperoxic pups, has a positive effect as shown in our long-term neuro- developmental studies (Figure 6). Similar role was shown after daily Galantamine administration in experimental traumatic brain injury, which showed significant improvements in cognitive deficits and histological recovery (Zhao et al., 2018).

Galantamine potentiates “cholinergic anti-inflammatory pathway.” This pathway inhibits cytokine release by neural signals, transmitted via the vagus nerve, through a mechanism that requires the alpha7 subunit-containing nicotinic acetylcholine receptor (α7nAChR). This reflex suppresses NF-κB activation and pro-inflammatory cytokine production (Tracey, 2002; de Jonge and Ulloa, 2007; Pavlov and Tracey, 2015). Therefore, in addition to inhibiting neuro inflammation, Galantamine can inhibit systemic inflammation. Since hyperoxia has deleterious effects on all organs in premature infants especially lung (Buczynski et al., 2013) and eyes, galantamine can ameliorate hyperoxia induced injury to those organs.

The reduced performance in open field exploration, novel object recognition in hyperoxic mice is likely a combination of damage to the cholinergic nuclei in addition to retinopathy and retinal degeneration. Galantamine ameliorated this retinopathy, likely contributing to the improved neurobehavioral outcomes. Further detailed studies need to be carried out to provide such evidence.

A limitation of this study is that our neonatal hyperoxia mouse model uses 95% oxygen for 7 days, while premature infants in the neonatal intensive care unit are sometimes exposed to much lower oxygen and still develop brain injury and poor neurodevelopmental outcome. We used this exaggerated hyperoxia condition, as proof of concept. Studies using 80%, even 65% oxygen are showing comparable results.

The results from our study are important as a preclinical model for controlled clinical trials in infants born very preterm. Their brains, even if ventilated with very high concentrations of oxygen, is never exposed to the very PaO_2_ of the experiments reported here, as FiO_2_ is titrated according to preductal arterial oxygen saturations using target ranges that are lower than normal values in healthy adults. Several large randomized controlled trials (SUPPORT, and BOOST-II) comparing higher versus lower target ranges of arterial oxygen saturation failed to find differences in neurodevelopmental outcome. However, A 3-4fold rise of PaO_2_ occurs in virtually all mammals after birth when the source of oxygen switches from the placenta to the lungs, and preterm infants are poorly equipped to deal with sudden and profound surges of oxygen.

In conclusion, premature infants are at high risk for hyperoxia leading to systemic inflammation and neuroinflammation. Activation of ‘the cholinergic anti-inflammatory pathway’ by galantamine administration can improve short- and long-term outcomes in this susceptible population. In addition, galantamine has potent anti-inflammatory and antioxidant effects in hyperoxia-induced neonatal brain injury. It decreases oligodendrocyte loss thus preserving myelination. Increasing cholinergic activity by galantamine improves neuronal survival and increases ChAT expression. Long term neurobehavioral studies show that when galantamine administration in hyperoxia improves locomotion, coordination, learning and memory. Our data suggest a prospective, innovative approach where galantamine could be clinically studied and applied as a new therapeutic agent in neonates with or at high risk for developing hyperoxia brain injury.

## Data availability statement

The raw data supporting the conclusions of this article will be made available by the authors, without undue reservation.

## Ethics statement

The animal study was reviewed and approved by Institutional Animal Care and Use Committee of the Feinstein Institute for Medical Research and the University of Arizona.

## Author contributions

NZ, NC, DK, KA, H-LL, and MA conceived the project and designed the experiments. NZ and NC wrote the manuscript. NZ, NC, KA, and MA performed all *in vivo* experiments. NZ, NC, and H-LL performed the immunostaining and all statistical analyses. KA performed the cytokine protein expression. NZ performed all the neurobehavioral testing. NZ, DK, H-LL, and MA revised the manuscript. All authors contributed to the article and approved the submitted version.

## Funding

NZ was supported by Department of Pediatrics, University of Arizona. University of Arizona Health Science CDA award given to NZ.

## Conflict of interest

The authors declare that the research was conducted in the absence of any commercial or financial relationships that could be construed as a potential conflict of interest.

## Publisher’s note

All claims expressed in this article are solely those of the authors and do not necessarily represent those of their affiliated organizations, or those of the publisher, the editors and the reviewers. Any product that may be evaluated in this article, or claim that may be made by its manufacturer, is not guaranteed or endorsed by the publisher.

## Figures and Tables

**Table 1:** 

Ach	Acetylcholine
AchE	Acetylcholine esterase enzyme
BFCS	Basal forebrain cholinergic system
CNPase	2',3'-Cyclic-nucleotide 3'-phosphodiesterase
ChAT	Choline acetyl transferase
CP	Cerebral palsy
DAMP	Damage Associated Molecular Patterns
DMN	Dorsal motor nucleus of the Vagus
LDT	Laterodorsal tegmental
Gal	Galantamine
GFAP	Glial fibrillary acidic protein
H_2_O_2_	Hydrogen peroxide
HMBG1	Brain high mobility group box 1
Iba1	Ionized calcium-binding adapter molecule 1
iNOS	Inducible nitric oxide synthetase
NA	Nucleus ambiguus
PPT	Pedunculopontin tegmentaltegmental
RA	Room air
ROS	Reactive oxygen species
WMI	White matter injury
