# Supplementary material for: Galantamine ameliorates hyperoxia-induced brain injury in neonatal mice
Source: Front Neurosci. 2023 Jun 22;17:890015. doi: 10.3389/fnins.2023.890015 (PMC10323435; doi:10.3389/fnins.2023.890015)
Supplement: Supplementary file 1 [file Data_Sheet_1.docx]

**Supplementary Materials**

**Material and Methods**

### TUNEL staining

A TUNEL Detection kit (GenScript, Pisactaway, NJ, USA) was used according to the manufacturer’s instructions. Briefly, after pretreatment of the slides with proteinase K, quenching of endogenous peroxidase, and rehydration with gradient of ethanol concentrations, slides were washed with PBS. FITC- labelled TUNEL reaction mixture was added to the slides, which were then incubated for 1hour. Then slides were rinsed in PBS, then slides were mounted, and cover slipped. Assay with a fluorescence microscope with an excitation wavelength of 450–500 nm and an emission wavelength of 515–565 nm was performed.

MRI instrumentation and data acquisition

At P60, MRI data were obtained using 9.4/30 BioSpect Spectrometer (Bruker BioSpin Corp., Germany) equipped with a 72-mm volume coil as a transmitter and a 4-channel mouse brain coil. High-resolution RARE T2-weighted images in axial and coronal plain were acquired with following parameters: TR/TE = 3524/36 ms, FOV = 1.5 cm, matrix = 256 *×* 256, 0.5 mm slice thickness, N slices = 30, Navg = 4, and RARE Factor = 8.

The animals were anesthetized on P60 using 2% isoflurane with carbogen (95%O2+5%CO2). Their respiration and temperature were monitored during the entire course of the experiment using Small Animal Instruments monitoring system (Small Animals Instruments, Inc., Stony Brook, NY, USA).

MRI data analysis

The axially acquired T2-weighted images (0.5 mm slice thickness) were used for data analysis. Mask of the mouse brain and hippocampi were obtained by manually tracing area of interest. Coronally acquired images were used for a conformation. Images were imported into Analyze 7.5 software (Biomedical Imaging Resource, Mayo Clinic, Rochester, MN) for manual and semi-automated volume rendering. We estimated brain volumes for the 0.5-mm gap using Analyze. *N* = 5 animals/group.

### Histopathological exam

Eyes were fixed on P14 in 4% paraformaldehyde for 24 h, processed, embedded in paraffin, and subsequently cut into 6-μm-thick sections. Following deparaffinization, hematoxylin and eosin (H&E) staining was performed according to standard protocols.

**Results**

H&E staining of cholinergic cells showed neuronal degeneration of the Lateral Dorsal Tegmental nuclei (LDT, upper panel) and Nucleus Ambiguus (NA, lower panel) in hyperoxic non-treated group (red arrows in Supplementary Fig.1). Galantamine treatment rescues cholinergic neurons from hyperoxia induced neuronal degeneration as shown in Supplementary Fig.1. There is no difference between RA+ saline and RA+ galantamine group.

To evaluate late stages of neuronal apoptosis and neuronal necrosis, we performed Tunel staining. We examined Tunel colocalization with ChAT positive cholinergic nuclei in the BFCS as an indication of cholinergic neuronal necrosis. Tunel staining was significantly increased in the hyperoxia BFCS ChAT positive nuclei as compared to the RA groups. Galantamine treated hyperoxia group had a significantly lower Tunel staining when compared to hyperoxia group, thus providing protection against cholinergic neuronal necrosis (Supplementary Fig.2).

Volumetric analysis using T2 weighted MRI was performed on P60. Total brain volumes showed no difference between the groups. Bilateral hippocampal volumes showed significant reduction in hyperoxia as compared to both RA groups. Galantamine treatment ameliorated this reduction in hippocampal volumes seen with hyperoxia. (Supplementary Fig. 3)

Hyperoxia can cause severe and permanent retinal degeneration. We show that in hyperoxia, the hyaloid artery is hyperplastic and extends onto the retina near the optic nerve head and forms a thick distinct capsule on the posterior lens surface. Outer retinal degeneration with elevated and disrupted retinal layers and neovascularization is evident in hyperoxic pups. In Galantamine treated pups that undergone hypreoxia, the hyaloid artery is less hyperplastic, also forms a capsule on the posterior lens surface and the outer retinal cell disruption and neovascularization is less severe. (Supplemental Fig.4)

**Figures**

**Figure Legends:**

**Supplementary Fig. 1**

H&E stain of the Laterodorsal Tegmental nuclei (LDT, upper panel) and Nucleus Ambiguus (NA, lower panel). Red arrows indicate degenerating cholinergic nuclei. Hyperoxia causes extensive neurodegeneration of cholinergic nuclei which is rescued by galantamine administration. Magnification 20x. Scaler bar= 100µm.

N = 5 animals/group & 4 sections/animal.

**Supplementary Fig. 2**

FITC-labelled Tunel detection kit shown in green and choline acetyltransferase containing cells (ChAT) labelled in red of the Basal forebrain cholinergic system (BFCS). Scale bar= 100 µm. N = 5 animals/group & 4 sections/animal. Quantification showing percent of Tunel+ ChAT + /ChAT + cells indicating percent of necrotic ChAT cells.

**Supplementary Fig. 3**

**(A)** Representative T_2_-weighted images of hippocampi at the Bregma -1.8mm in all 4 groups at P60. **(B)** Total brain volume showing no difference between the groups. **(C)** Bilateral hippocampal volume showing significant differences between the groups. Scatter dot plot showing mean ± SE. P ns, **P< 0.01, ***P<0.001, ****P≤ 0.0001. N=5mice/group.

**Supplementary Fig. 4**

Histopathology H&E of representative eyes on P14.

Normal anterior segment morphology, lens and vitreous of RA **(A)** and RA +Galantamine**(B).** Normal retina histology in RA **(E)** and RA +Galantamine **(F).** In hyperoxia, the hyaloid artery (yellow arrow) is hyperplastic and extends onto the retina near the optic nerve head and forms a thick distinct capsule on the posterior lens surface **(C)** that extends laterally and appears to send branches to supply the peripheral retina (green arrow). Outer retinal degeneration with elevated and disrupted retinal layers with evidence of neovascularization **(G)** as well as neovascularization over the optic nerve **(I).**  In Hyperoxia +Galantamine, a hyaloid artery s hyperplastic and extends on the posterior lens surface **(D).** Outer retinal disruption **(H)** but less severe than hyperoxia. Neovascularization along the posterior lens surface **(J)** but less severe when compared to hyperoxia.

*Magnifications*: 10x for A-D, 50x for E-H and 100x for I, J.
