# Supplementary figures and images for: Galantamine ameliorates hyperoxia-induced brain injury in neonatal mice
Source: Front Neurosci. 2023 Jun 22;17:890015. doi: 10.3389/fnins.2023.890015 (PMC10323435; doi:10.3389/fnins.2023.890015)

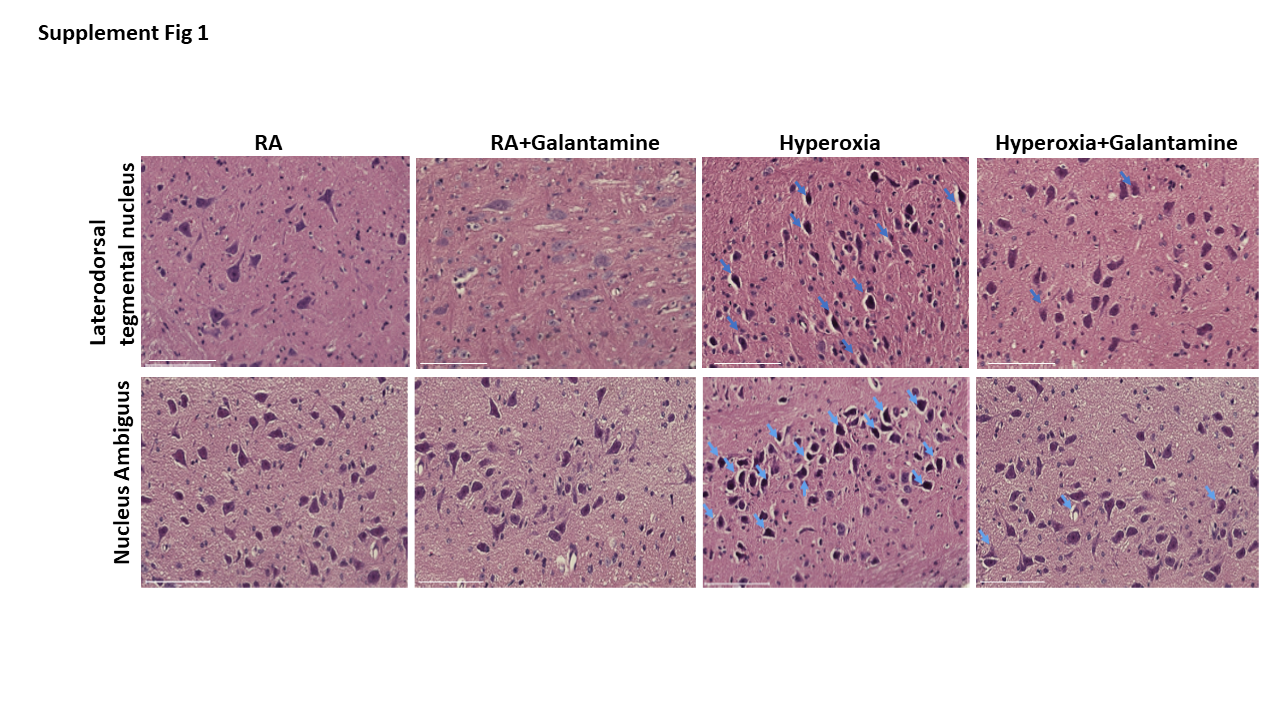

Supplement: Supplementary file 2 [file Image_1.TIF]

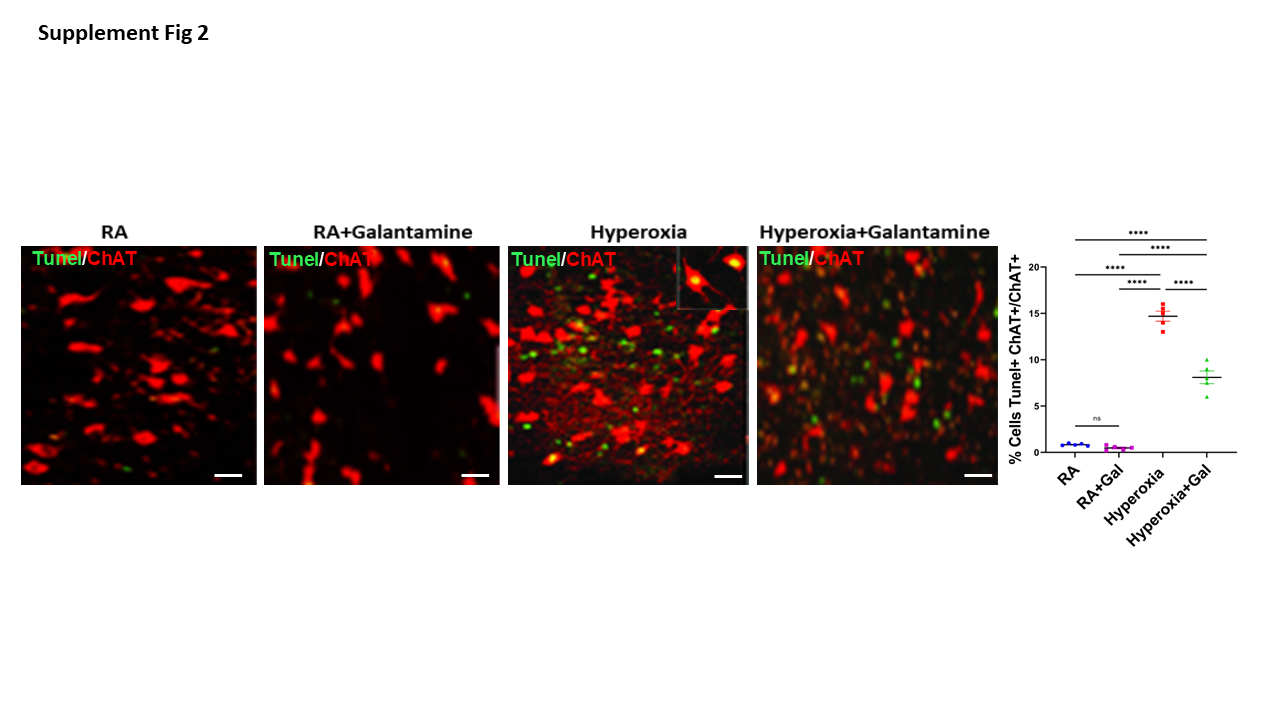

Supplement: Supplementary file 3 [file Image_2.TIF]

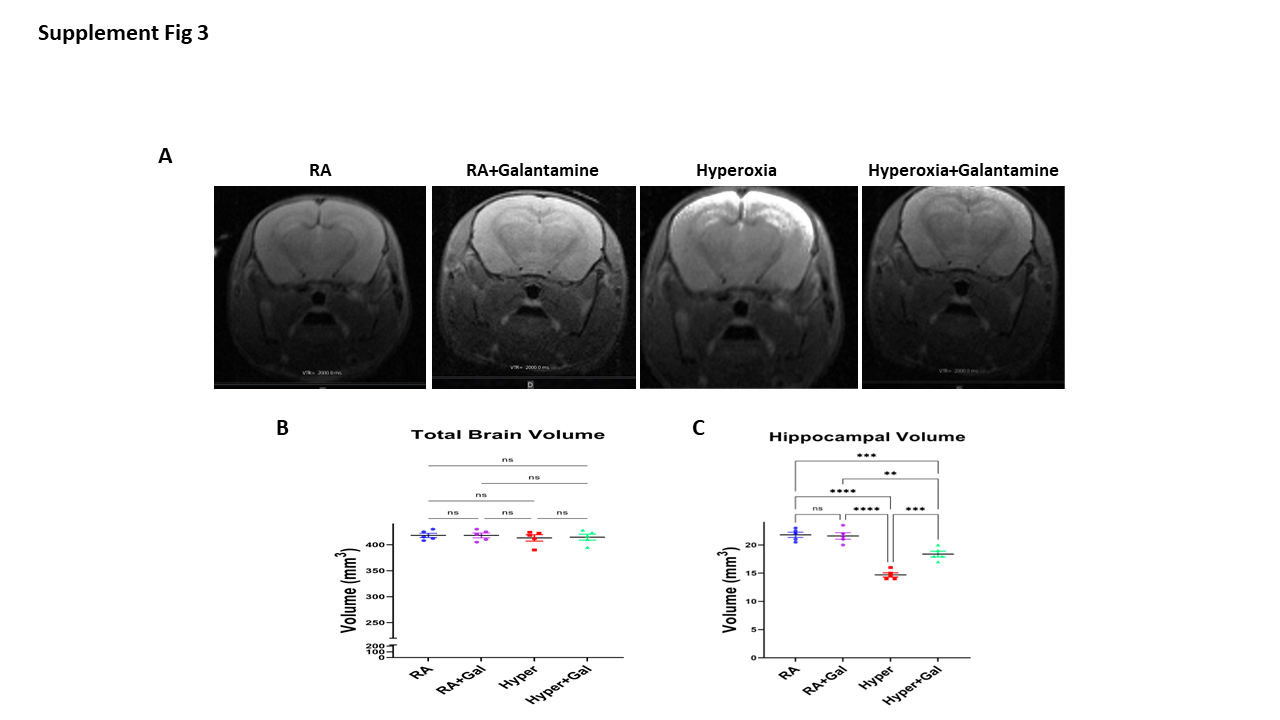

Supplement: Supplementary file 4 [file Image_3.TIF]

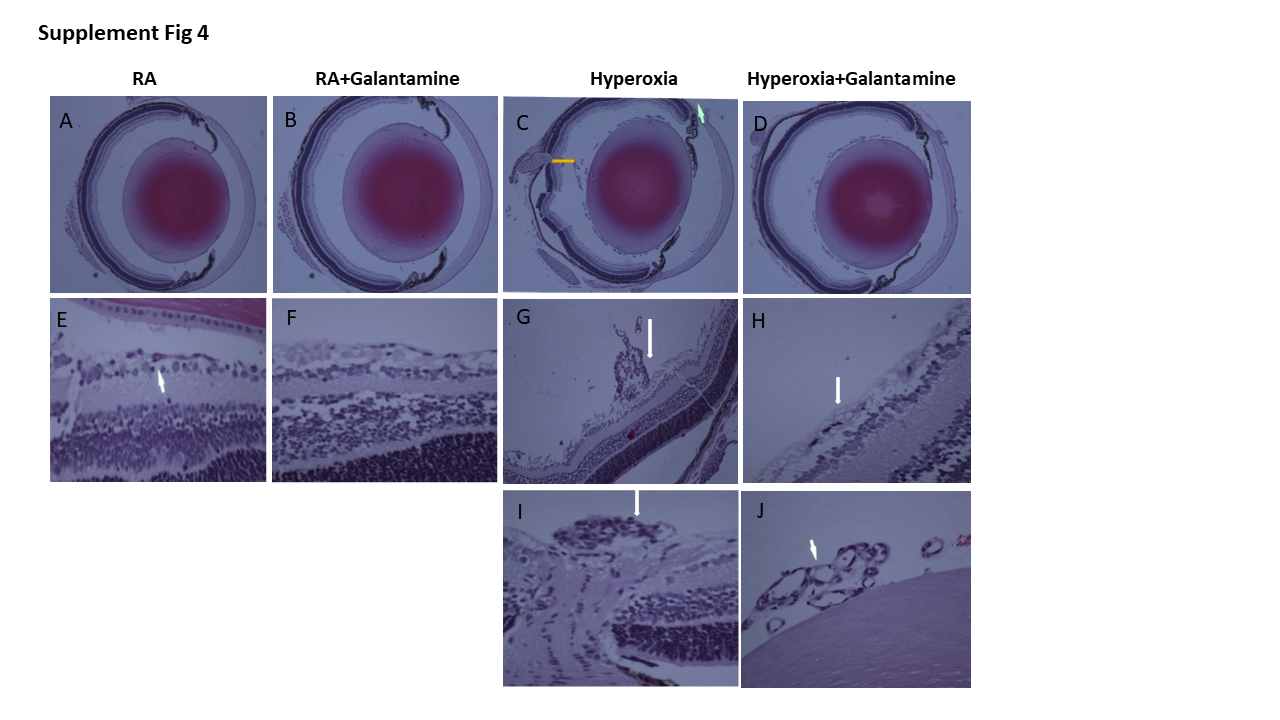

Supplement: Supplementary file 5 [file Image_4.TIF]
